# Combining Nanotechnology and Gas Plasma as an Emerging Platform for Cancer Therapy: Mechanism and Therapeutic Implication

**DOI:** 10.1155/2021/2990326

**Published:** 2021-10-27

**Authors:** Milad Rasouli, Nadia Fallah, Sander Bekeschus

**Affiliations:** ^1^Plasma Medicine Group, Endocrinology and Metabolism Research Center, Endocrinology and Metabolism Clinical Sciences Institute, Tehran University of Medical Sciences, Jalale-Al-Ahmad Ave, 1411713137 Tehran, Iran; ^2^Department of Physics and Institute for Plasma Research, Kharazmi University, 49 Dr. Mofatteh Ave, Tehran 15614, Iran; ^3^Department of Cell and Molecular Biology, Faculty of Biological Sciences, Kharazmi University, 49 Dr. Mofatteh Ave, 31979-37551 Tehran, Iran; ^4^ZIK Plasmatis, Leibniz Institute for Plasma Science and Technology (INP), Felix-Hausdorff-Str. 2, 17489 Greifswald, Germany

## Abstract

Nanomedicine and plasma medicine are innovative and multidisciplinary research fields aiming to employ nanotechnology and gas plasma to improve health-related treatments. Especially cancer treatment has been in the focus of both approaches because clinical response rates with traditional methods that remain improvable for many types of tumor entities. Here, we discuss the recent progress of nanotechnology and gas plasma independently as well as in the concomitant modality of nanoplasma as multimodal platforms with unique capabilities for addressing various therapeutic issues in oncological research. The main features, delivery vehicles, and nexus between reactivity and therapeutic outcomes of nanoparticles and the processes, efficacy, and mechanisms of gas plasma are examined. Especially that the unique feature of gas plasma technology, the local and temporally controlled deposition of a plethora of reactive oxygen, and nitrogen species released simultaneously might be a suitable additive treatment to the use of systemic nanotechnology therapy approaches. Finally, we focus on the convergence of plasma and nanotechnology to provide a suitable strategy that may lead to the required therapeutic outcomes.

## 1. Introduction

Albeit progress continues, cancer remains a devastating disease in millions of patients worldwide. In 2020, over 19 million new cancer cases are projected to occur globally [1]. The standard treatments for cancer therapy include radiotherapy, chemotherapy, surgery, and immunotherapy. These therapeutic strategies yield inadequate therapeutic efficacy in some patients or have unfavorable safety profiles [2]. Another challenge of current treatment methods is the therapy resistance related to tumor cells' intrinsic or acquired exit strategies to circumvent cytotoxic therapy effects [3]. For instance, tumors consistently comprise a mixture of drug-sensitive cells and stem cells, which leads to adaption and drug resistance [4]. Hence, efforts have been dedicated to exploiting multimodal, flexible, and multifunctional therapeutic modalities. In combination with main treatment strategies, nanomedicine helps overcome numerous oncotherapy obstacles and might reduce side effects for enhancing treatment tolerability of conventional treatment [5].

Nanotechnology refers to different designs of matter in nanoscale. This technology has emerged as a multidisciplinary scientific field, including physics, chemistry, engineering, and biology. In recent years, nanotechnology has gained much attention, especially in medicine, known as nanomedicine [6]. Nanoparticles (NPs) are utilized to treat, diagnose, image, and prevent disease spread in cancer. Different types of NPs with several properties, such as drug delivery, have been created to complement current treatments. Organic NPs (e.g., lipid-based and polymeric NPs) and inorganic NPs (e.g., silica NPs and quantum dots), or the combination of them, indicate an efficient oncotherapy by targeting solid tumors [7]. Improved drug solubility, stability in the bloodstream, target delivery to tumors, control released, and reduction in toxicity are the outstanding features that distinguish this strategy from other therapies. Besides, enhancement in permeability and retention is accompanied by a high accumulation of NPs in tumors compared to normal tissues [8].

Gas plasma, produced at body temperature by applying an electric field to one or a set of electrodes, represents a multimodal environment of physical and chemical factors [9]. This technology has introduced an exciting application to modern medicine, ranging from wound healing, decontamination and antiviral action, and surface modification to recently also cancer therapy [10, 11]. Plasma cancer therapy is one of the most investigated applications of this technology today by engaging multiple disciplines, including engineering, physics, biology, and medicine, to achieve a novel oncotherapeutic approach. With effective targeting of multiple cancer hallmarks, gas plasmas provide a cocktail of physicochemical agents having great potential for translational cancer medicine separately or in combinatorial use with conventional therapeutic modalities [12]. Gas plasma treatment is performed directly by bringing the target tissue in immediate contact with the plasma plume or indirectly by exposing liquids suitable for clinical practice [13]. At the level of preclinical studies, gas plasma treatment showed a selective antitumor action to some extent [14], improves combination chemotherapy [15], and inhibits metastatic spread [16]. It is understood that these actions result from the multi-ROS/RNS (reactive oxygen species/reactive nitrogen species) generation by gas plasmas [17]. Apart from this, gas plasma can be combined with conventional therapies [18] due to its adjustable and flexible properties [19]. This introduces gas plasma as a promising modality in cancer treatment, separately or in combination with conventional methods and new technologies.

In pursuit of an innovative oncotherapeutic strategy, the combination of nanoparticles and gas plasma with their main features is presented. Moreover, therapeutic outcomes, efficacy, and implication of each technology are being discussed. To advance cancer treatment modality development, the convergence of plasma and nanotechnology in oncology, especially the nexus between reactivity and therapeutic implications of these therapeutic modalities, is summarized. The future horizons with opportunities and challenges also are presented.

## 2. Nanotechnology as a Platform for Oncotherapy: Types of Material and Targeting Systems

Playing a significant role in the COVID-19 vaccine development [20], nanotechnology was once again introduced as a multifunctional platform in resolving healthcare-related challenges. Cancer nanomedicine, which utilized nanotechnology for combating cancer, received significant attention owing to the promising results. Here, we present the NPs used to treat cancer based on their main features. Further, with a particular focus on NPs delivery vehicles, the therapeutic implications are described in detail.

### 2.1. Main Features of Appropriate Nanoparticles for Cancer Treatment

By their tunable capacity for loading agents and the facilitation and accuracy in drug delivery [21], nanocarriers are proper candidates for experiments at the level of in vitro and in vivo research and clinical trials [22]. In general, the use of NPs, due to their properties in various cancers, might play an essential role in the effectiveness of treatment across biological barriers. Charge, hydrophobicity, and surface cloaking are the surface properties of NPs, and shape, size, elasticity, and porosity are their physical features. Altering these physicochemical properties is the changes the subsequent penetration and toxicity profiles of NPs [23].

The surface coating, shapes, size, and elasticity of NPs play crucial roles in their biodistribution and pharmacokinetics in clinical and preclinical experiments [24–26]. Besides, the rate of internalization is linked to the shape and size of NPs [27]. Spherical NPs are very common and gained trust during these years. However, nonspherical properties with their unique characteristics gained attention in recent years [28]. It is interesting to note that the shape of the NPs is more important for the attraction of macrophages and phagocytosis than their size [29]. Evidence suggests the deviating hydrodynamic manner of nonspheroidal NPs; so ,their circulation time in the blood is more extended than spheroid NPs [30]. The aggregation of NPs at tumors sites is regulated by their shape, too [31]. The size of NPs is directly related to their effectiveness and biological function in experiments. Further, the formation of nanocarriers and agents is affected by the NP size [32]. Regarding elasticity, soft NPs represent higher permanence in blood circulation compared to hard NPs. On the contrary, hard NPs demonstrate higher cellular uptake rates. Accordingly, soft and hard NPs, according to the type of organ, display varying distributions [33].

NPs can have a positive or negative surface charge based on different components employed during their production. The surface charge has a significant effect on the stability, encapsulation capacity, and biodistribution of NPs. For instance, a slightly negative charge causes a better accumulation of NPs in tumor tissue [34]. Surface hydrophobicity has an essential role in immune processing and phagocytosis through opsonization and quicker blood clearance. Nowadays, using PEGylation (covalent or noncovalent attachment of amalgamation of polyethylene-glycol for masking an agent to reduce antigenicity) and hiding surface charge and hydrophobicity enhances the durability of NPs in blood circulation [31, 35]. In addition to PEG, some other factors for NP coating include peptides and biological membranes for concealing NPs and giving them unique properties [36, 37]. Hence, the active targeting decrements toxic effects in nonmalignant cells and enhances cellular uptake of NP-based drugs in tumors.

NPs in drug delivery systems for oncotherapy can be coated with different organic or inorganic substances containing, for instance, metals, polymers, carbon, lipids, and proteins. These NPs, based on their hydrophilic or hydrophobic properties, also can encapsulate different agents. For example, liposomes with their hydrophilic core are suitable for hydrophobic therapeutic compounds [38]. Concerning polymeric NPs, different types of polymers (synthetic or natural) with biocompatible and biodegradable properties are used for drug delivery. Emulsion polymerization, emulsion evaporation, emulsion diffusion, nanoprecipitation, salting-out, dialysis, and supercritical fluids are used for synthesizing polymeric NPs [39]. In recent decades, metal-based (inorganic) NPs made of gold, silver, superparamagnetic iron oxide, and quantum dots have been utilized for experimental therapy and especially tumor diagnosis [40, 41].

### 2.2. Nanoparticle Delivery Vehicles

NPs should have specific properties for the successful delivery of therapeutic agents to tumor tissue. First, NPs require a particular marker or antibody targeted against tumor cells to reduce side effects to nonmalignant tissues. All types of NPs, including micelles, liposomes, and polymeric NPs, can load antibodies on their surface to increase efficacy and improve clinical trials' outcomes [42]. However, leakage of blood vessels and insufficient lymphatic drainage often result in drugs not reaching tumor cells sufficiently. Hence, targeted NP therapy is a suitable strategy to prevail these obstacles in tumor cells [8]. For instance, iron oxide NPs linked to anti-CD44 monoclonal antibodies are utilized for cancer cells with the high CD44 expression [43]. Polymeric and magnetic NPs coated with anti-HER2 antibodies are used for HER2-receptor-positive cancers, especially ovarian and breast cancer [44, 45]. Transferrin-coated liposomes are used against head and neck cancer [46] and glioblastoma [47].

At the same time, the immunological dimension of cancer therapy is increasingly being recognized, as evident by the advent and success of immunotherapies in the 21^st^ century [48–50]. Therefore, NPs have been heavily investigated in the past decade for their effects of providing and stimulating antitumor immunity in several types of cancer. Notably, the versatility of NPs lies in their tunable composition and hence target penetration and delivery, as recently summarized for macrophage update [51]. As another example, NPs were shown to perform targeted delivery of miR-200c and a CXCR-4 antagonistic peptide that led to immunogenic cancer cell death (ICD), perpetuating antitumor immunity, decreasing immunosuppression, and abrogating the expression of immune checkpoints in the tumor microenvironment [52]. Primarily gold nanoparticles are envisioned to perform a dual role as immune regulators and drug delivery into the tumor tissue [53]. NPs were recently proposed as efficient vehicles for anticancer vaccines, owing to their unique properties in targeted delivery and tissue penetration [54]. Nevertheless, care must be taken that NPs do not overstimulate immunity, leading to multiple organ failures. Along those lines, other safety aspects need to be considered, including NP reactions with proteins in the blood, nonphysiological activation of platelets leading to coagulopathies, excessive cellular damage, and hemolysis [55]. By crossing biological barriers, some NPs can cause adverse effects on various organs kidney, liver, brain, and reproductive systems. For instance, aggregation of NPs in the reproductive system by toxicity inducing impair the cells related to reproductive function. Although the exact molecular mechanisms and signaling are not clear, apoptosis, stress oxidative, and inflammation are among the response of these organs to NP toxicity [56]. AgNPs are widely used for antimicrobial properties in medicine, but this kind of NP can cause alteration in neurobehavioral and organ development in offspring after long-term exposure. AgNPs passing the blood-brain barrier (BBB) and disrupting development in the fetal brain can induce oxidative stress causing sensitivity against infection [57].

The last factor for drug delivery in oncotherapy is controlled drug release, which some of the elements used for NP generation can regulate. The purpose of the controlled release of drugs from NPs is to preserve the drug coating during the NP journey in the bloodstream and increase its toxic effect once delivered to the purpose destination in the tumor microenvironment (TME) [58]. In general, it should be noted that the optimal concentrations are achieved after an appropriate dose is applied that allows maximum tumor toxicity while retaining acceptable levels of side effects [59]. Stimuli-responsive NPs for drug release are categorized into two groups responsive to either internal and external stimuli. For example, pH, temperature, electric field, magnetic fields, and glutathione levels are used as stimuli [60–62]. Moreover, polymeric NPs can release agents by a hydrolytic or enzymatic method called degradation-controlled release. In this strategy, bonds in the backbone of NPs are being destructed for triggering drug release [63]. The solvent-controlled release, which works based on osmosis or swelling, is another method for releasing drugs from NPs. The osmosis-controlled release is suitable for NPs with semipenetrable membranes [64], while swelling-controlled release occurs in polymeric NPs with a glassy hydrophilic membrane [65]. In the latter, water can quickly enter the NPs present in, for instance, hydrogels, and there is a direct relation between the rate of water diffusion and drug release.

### 2.3. Therapeutic Outcomes of NPs in Oncology

One of the essential applications of NPs is their targeted delivery of agents for oncotherapy engineered according to the type of cancer and the therapeutic agents, as well as the unique properties of the nanoparticles (Table 1). Overall, the use of NPs for the treatment or diagnosis of cancer is not limited to preclinical experiments. There have been many successes in clinical trials in several cancer entities; albeit, approval for medical use still is awaited in many instances. Colorectal cancer, breast cancer, melanoma, and head and neck cancer are examples of using NPs in clinical trials [66]. Gold nanoparticles (AuNPs) induce toxicity in tumor cells, and their size is directly associated with the rate of penetrance, leading to toxicity effect by increasing ROS/RNS levels and subsequently induce oxidative stress. AuNPs are also used for imaging and probing tumor tissues. This is facilitated by free electrons of gold atoms being exposed to light, which leads to collective oscillation, also known as localized surface plasmon resonance, and subsequent light emission. Moreover, especially smaller AuNPs transmute light to heat and, as a result, are suitable for photothermal therapy [67, 68]. Due to their simple synthesis, suitable pharmacokinetics, and low toxicity profile, gold nanoparticles have drawn significant attention in the field of cancer therapy in recent years [69, 70].

Quantum dots are known as semiconductor NPs, and their characteristics originate from their ability to scatter fluorescent light from the visible to the infrared spectrum after excitation [71]. Quantum dots can help image small tumors in their initial stage that are otherwise difficult to diagnose [72]. Moreover, to better recognize tumor cells, they can be conjugate to different types of antibodies on their surface, which helps increase their utilization in clinical trials [73]. Polymeric-based NPs generally are made from naturally degradable materials such as polysaccharides, chitosan, hyaluronic acid, alginates, dextran, protein-based polymers, collagen, gelatin, and albumin, which do not cause toxic effects in the human body but can exert antitumor effects based on their cargo [39]. As an example of polymeric NPs, hyaluronic acid can affect tumor cell proliferation and angiogenesis, while albumin NPs can penetrate the blood-brain barrier. Chitosan NPs, by their unique features, have an essential role in tumor growth inhibition and apoptosis induction [74].

Lipid-based NPs consist of natural hydrocarbons or are being derived from plants and animal material. They can also be composed of synthetic phospholipids, cholesterol for membrane bilayer, and sphingolipids. For increasing therapy efficacy, lipid-based NPs can be conjugate with polymeric residues such as PEG and PEI (polyethyleneimine). Their form is usually spherical, and by active targeting, they enhance the drug's pharmacodynamics and pharmacokinetic properties [75, 76]. Lipid-based NPs can inhibit migration and invasion of tumor cells and improve the internalization of anticancer drugs loaded on lipid-based NPs compared to free drugs [77]. Mesoporous silica NPs are another widely used type of NPs, having a high capacity for encapsulating therapeutic agents and showing adjustable drug release. They are also utilized for optical imaging, ultrasound and magnetic resonance imaging, and positron emission tomography [78]. Furthermore, the alterable pore size of mesoporous silica NPs makes them a good option for proteins transfer [79]. Besides, they can easily be decorated with different small molecules including folate, transferrin, VEGF, IGF, EGF, C-type lectin, mannose, asialoglycoprotein, and monoclonal antibodies targeting, for instance, HER2, CD44, TLR9, and integrins as a marker to improve the detection of cancer cells [80]. Ultimately, this can lead to decreased tumor volumes owing to enhanced cellular uptake of the NPs.

The mainstay of future clinical cancer treatment is combination therapy between novel technologies and conventional strategies. Nanomedicine and gas plasma as documented oncotherapeutic modalities have great potential to potentially improve cancer treatment due to the multifunctional capacity of NPs and the multimodal nature of gas plasmas.

## 3. Plasma Oncology: Processes, Efficacy, and Mechanisms of Action and Challenges

Medical gas plasma technology, also known as cold physical plasma, is a partially ionized gas generated at atmospheric pressure and operated at body temperature. It is distinguished for generating a complex physicochemical flux of agents, including ions, electrons, mild thermal radiation, UV light, electric fields, and ROS/RNS [81]. The latter has been identified as unique agents to deliver the biotherapeutic effects [82]. While plasmas generate a mixture of ROS/RNS simultaneously with defined spatiotemporal profiles [83, 84], the overall deposition of these redox agents can be controlled either via the treatment time or energy in put [85]. Once close to biological targets, the ROS/RNS react with different biomolecules and partially oxidize, for instance, proteins [86], peptides [87], amino acids [88], lipids [89], and nuclei acids [90]. Accordingly, gas plasma-treated cells are potentially challenged by multiple ways, including diffusion of long-lived ROS such as hydrogen peroxide into the cytosol via aquaporins [91], lipid peroxidation [92], uptake of proteins with oxidative posttranslational modifications (PTMs) [86], and stresses through damage-associated pattern (DAMPs) being released into the microenvironment [93]. Due to the apolar nature of cell membranes, it is unlikely that the majority of species will enter the cytosol, as most ROS/RNS will find plentiful reaction partners at cellular membranes and their immediate vicinity to react with [17].

### 3.1. Gas Plasma Generation and Delivery Technologies

Gas plasma is generated by electric discharges and represents a partly ionized gas, where all heavy particles except electrons remain cold. The collisions between surrounding air and gas plasma-derived species bring about a physicochemical environment, which comprises the reactive agents including ROS/RNS. Depending on the different device geometries (plasma jet, dielectric barrier discharge, and plasma torch) as well as device configurations and parameters along with individual treatment procedures, different amounts of reactive compounds are being produced, leading to different intensities of the effects observed [94].

Plasma treatment is the process of transferring a set of physical and chemical agents to the target. An important consideration, and perhaps downfall, of the field of plasma medicine is the polypragmasia in the use of plasma devices. Hundreds of different plasma sources for biomedical application have been published, and most work is not necessarily building on top of previous knowledge but is instead reproduced based on methods in physics, chemistry, and cell biology. A clear scheme on optimal plasma source design considerations and technical parameters is not present. However, several sources have been developed in Germany; among them, the first true (cold) medical gas plasma devices intended for medically accredited use in dermatology centers in Europe [95]. Notwithstanding, it is understood that despite different geometries and ROS/RNS profiles, gas plasma treatment overall produces similar effects, being stimulating at low doses, treatment times, or energy input, and toxic at higher doses, treatment times, or energy input as predicted by the concept of hormesis [82].

Direct plasma treatment and gas plasma-treated solution (PTS) are two very different plasma treatment procedures. Direct treatment transfers all physical and chemical agents concomitantly on target, especially the short-lived ROS/RNS unique to the gas plasma technology. When treating a liquid, some of the species can be retained in such liquid and stored for later therapeutic use. This concept is called plasma-oxidized liquids (POL) that can be used for clinical application if using solutions certified as medical products such as sodium chloride [13]. Alternative names for the concept are plasma-treated liquids (PTL), plasma-treated solution or saline (PTS), plasma-activated medium (PAM), and plasma-activated liquid (PAL), among others [96]. POS recently has received significant attention in widespread areas, especially where direct plasma treatment has faced challenges. Several animal models have shown the versatility of POL [97–99]. Current challenges include its large bulk liquid generation, storage, sterility, and the lack of animal studies showing a benefit of such liquids over concentration-matched hydrogen peroxide solutions.

### 3.2. Cocktail of Physical and Chemical Factors in Gas Plasmas

ROS/RNS are produced in several stages based on plasma interaction with air, liquid, and matter and appear to play a vital role in the plasma therapy process [94]. The most important aspect of plasma differentiation, along with the diversity of physical and chemical factors and their combination, is their controlled and adjustable transfer to the biological target. Thus, depending on the input factors (e.g., discharge voltage, external electric field, target capacitance above ground, gas flow rate, and quenching gas shielding), a specific concentration of ROS/RNS is generated, which is not achievable in any other conventional cancer treatment methods [17], including photodynamic therapy. At the same time, UV and microwave emissions', positive ions, and electrons as main output parameters are highly related to the input parameters; albeit, their individual contribution to anticancer effects has not been studied so far, primarily because of the lack of ability to separate such factors from the ubiquitous ROS/RNS being generated simultaneously. For more detail regarding the nexus between the inputs and output parameters, see [100].

Apart from identifying the chemistry being critical for biomedical gas plasma effects, the short half-life of generated ROS/RNS [101] and the low penetration depth of species in cells and tissues [102] remain a practical challenge in some applications. For example, the half-life of hydrogen peroxide, nitrite, nitrate, and ozone is on minutes to hours scale, depending on the temperature, whereas for other species such as atomic oxygen, hydroxyl, and nitric oxide, it varies between nanoseconds to seconds [103–105]. Although it has been reported that gas plasma triggers tissues effects in cm ranges, it has to be kept in mind, however, that the penetration depth of the most reactive species is about a few micrometers only, which is not enough to penetrate the tissue and seems appropriate for superficial skin lesions treatment. Notwithstanding, the signaling function of these gas plasma-derived ROS/RNS seems to transport information deep into tissues, as demonstrated using hyperspectral imaging of murine gas plasma-treated skin and wounds [106–108]. Hence, the current model is that superficial layers are being oxidized by the gas plasma-generated ROS/RNS, subsequently leading to PTMs and oxPTMs (oxidative post-translational modifications) on biomolecules, ultimately being sensed by cells and translated into differential signaling responses [109]. OxPTMs are increasingly recognized as signaling agents in, for example, neurodegenerative and cardiovascular disease [110–112]. Oxidative distress occurs at supraphysiological ROS/RNS concentrations, and cell and tissue damage may be induced directly [113]. The biological responses can then affect neighboring cells via paracrine routes via soluble factors or communication via junctional proteins to deeper layers of the tissue [107, 114].

The other physiochemical parameters of gas plasma are thought to play a minor role. UV radiation is present but relatively weak [115]. Electric fields are moderate with dielectric barrier discharges [116] and helium plasma jets [117] and weak for the clinically relevant argon plasma jet kINPen, but the fields on their own cannot recapitulate the plasma effect.

### 3.3. Anticancer Effects and Mechanisms of Gas Plasma Therapy

Even though significant progress has been achieved in recent years, the exact dose definition and optimization of plasma devices remain a debate due to the variety of plasma devices, different therapeutic procedures, and input factors affecting the composition of the produced plasma. Primarily, the concentration of produced ROS/RNS is considered the plasma dose, and based on that, the effect of gas plasma on cancer cells is classified in the majority of cases as programmed cell death as evident in vitro [17], in vivo [93], in ovo [118], and ex vivo in human patient samples [119].

At low doses, gas plasma exposure causes autophagy, senescence, and cell cycle arrest. Concomitant modality of gas plasma and silymarin nanoemulsion (SN) resulted in autophagy activation in human melanoma cells (G-361) [120]. Besides, it was reported that the cell viability of AMEC and HEC50 cells, relevant to endometrial cancer, was decreased through POL treatment, and this was related to the induction of autophagic cell death [121]. Furthermore, short gas plasma exposure led to a senescence phenotype in the adipose-derived stromal cells (ASC) and dermal fibroblasts [122]. Senescence induction was also found in melanoma cells following gas plasma exposure [123]. This was found to be related to calcium influx [124]. Simultaneously, several studies showed that gas plasma treatment induces cell cycle arrest. Lung adenocarcinoma (A549 cells), epidermal papilloma (308 cells), glioblastoma (U87MG cells), epidermal carcinoma (PAM212 cells), and wild-type keratinocytes are among the reported cell line that gas plasma able to induce cell cycle arrest in them, especially at G2/M and G1/S and checkpoints [15, 125, 126].

Regardless of the various affected signaling, apoptosis is the most documented type of cell death that has been evaluated following gas plasma treatment. It can be claimed that the induction of apoptosis has been shown in the majority of cancer types that have been studied yet by gas plasma and POL. For example, we recently indicated that POL with high selectivity induces intrinsic apoptosis in chemotherapy-resistant ovarian cancer cells accompanied by high expression of p53, Bax, and caspase-3 [127]. Overall, in moderate concentrations of ROS/RNS, apoptosis is induced by gas plasma exposure.

Interestingly, gas plasma can induce ICD, a type of cell death eliciting an immune response that is highly important in progress on plasma oncology [128]. To overcome the penetrating depth challenges of gas plasmas-generated ROS/RNS into tumors, inducing ICD by gas plasma is the milestone of this multidisciplinary technology to introduce gas plasma as an emerging approach to complement traditional and novel oncotherapeutic modalities such as immunotherapies. This was previously shown in a vaccination model in mice [129] and in a model of elevating protein immunogenicity in a melanoma model [86]. The immune-stimulating effects of gas plasma were very recently shown to be dramatic, showing direct evidence of abscopal effects in a syngenic breast cancer tumor model in vivo [93]. Such effects are observed at high treatment energies or long exposure times, while low energy and short treatment times were also documented to be beneficial for tissue regeneration, including proangiogenic, and wound healing effects [130].

Further, nonprogrammed cell death might occur under a high dose of ROS/RNS so that both normal and cancer cells are affected and might cause undesirable hallmark effects and tissue damage. Therefore, the concentration of ROS/RNS should be adjusted for acquiring a unique environment for oncotherapy through gas plasma. Besides the abovementioned therapeutic efficacies, selectivity towards cancer and normal cells has been described in some reports [131–133]. The selectivity mechanism has been ascribed to the chemistry of gas plasma and the fundamental difference between cancer and healthy cells. Briefly, the high ROS/RNS baseline, more abundant aquaporins in cell membranes, and the lower cholesterol content in cancer cells versus healthy counterparts form the basis of selectivity [134–137].

It is important to note that several end-stage head and neck cancer patients have benefited from clinical gas plasma treatment using the medically accredited atmospheric pressure argon plasma jet kINPen MED [138]. Tissue analysis revealed induction of apoptosis but not severe side effects [139, 140]. Based on the results and responding vs. nonresponding patients, it was hypothesized that the immune system might have contributed to the therapeutic effects observed [141].

## 4. Future Horizons: Convergence of Plasma and Nanotechnology in Oncology

Although the combination of nanotechnology and gas plasma is still in preclinical settings, the results show a new strategy that in the future might be able to advance the efficacy of conventional therapies. Here, we emphasize the complementary role of plasma and nanotechnology technologies in improving each other's performance and highlight their main features that led to promising results. In addition, the nexus between reactivity and their therapeutic implications and potential challenges for translating into clinical uses are presented as the basis for a future innovative trend in cancer treatment.

### 4.1. How Nanotechnology and Gas Plasma Complement Each Other

Considering the multifunctional and practical properties of nanotechnology platforms and gas plasmas in addressing various cancer hallmarks, the combination or concomitant modality of these technologies arises an emerging strategy towards personalized medicine for cancer patients [142]. Albeit advances in nanomedicine are more than plasma oncotherapy, gas plasmas with promising outcomes led to the emergence of multimodal, safe, and controllable therapy for cancer treatment. While the complexity of tumor morphology on the one hand and the toxicity of some NPs on the other are the biggest challenges of nanomedicine in cancer therapy, the low penetration of gas plasma-produced ROS/RNS and the complexity of controlling and determining gas plasmas' dose are the essential troubles in plasma cancer therapy [143, 144].

**Figure 1:**
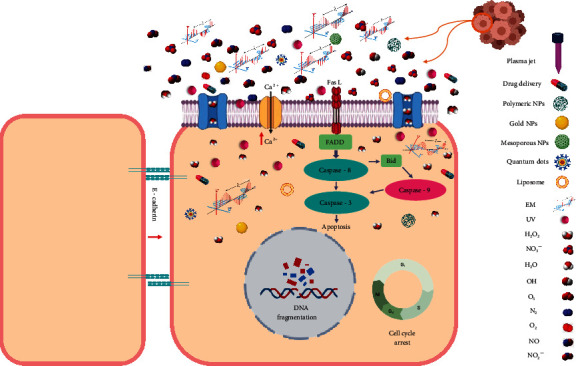
Combinational use of nanoparticles and gas plasma for cancer treatment, where reactive oxygen and nitrogen species along with the gas plasma-derived electromagnetic field and UV radiation affect tumor cells through the membrane. Reducing the pH acts as a complementary agent for improving nanoparticles efficacy and reducing toxicity. In addition, nanoparticles as carriers facilitate transferring gas plasma-generated reactive species to deep biological targets and may moderate the gas plasma irradiation time in highly selective ranges.

The proposed synergy of gas plasma and NPs is such that in addition to improving each other's strengths, they also cover each other's limitations (Figure 1). NPs have great potential to combine locally with gas plasma-generated ROS/RNS [145]. Moreover, the combinational use of gas plasma with NPs might reduce the minimum NPs concentration required, decreasing NPs toxicity as a novel strategy. Gas plasma improves the delivery of NPs and increases ROS/RNS in the target tissue. Such mechanisms have been previously shown in gas plasma-treated murine and human skin [107, 146–148]. Moreover, one of the most promising applications of these two technologies is the combination with chemotherapy drugs [149–151]. The potential combinatory routes are numerous and include, for instance, extrinsic and intrinsic apoptosis, enhanced drug transporter activity, DNA damage, oxidative stress, mitochondrial membrane collapse, growth factor deprival, and enhanced immune cell activity. Interestingly, in dermal applications, plasma appears to facilitate the penetration of NPs into the upper layers of the skin, and one hypothesis is that this is based on the plasma-generated electric fields [152]. In particular, transdermal delivery is an exciting field for combining plasma and nanotechnology, where the plasma-derived electric field is the most crucial factor for the efficient transfer of biological materials such as proteins, NPs, dextrans, and liposomes [153–155].

Due to the peroxynitrite production, gas plasma reduces the pH of tissue fluid or tissue in a rapid and reversible process and creates the acidic conditions required for the delivery of NPs [156, 157]. Another mechanism affecting the delivery NPs is the localized variation of temperature as adjuvant treatment in preclinical studies [158]. We have recently observed that the combination of hyperthermia with gas plasma leads to encouraging results for melanoma cancer treatment (unpublished observation). Therefore, the combination of hyperthermia, gas plasma, and nanomedicine seems to lead to an innovative combination therapy by increasing membrane fluidity, reducing tissue pH, and targeted transfer. Furthermore, the electric fields generated by the plasma possibly improve the magnetic NPs' performance for cancer therapy.

### 4.2. Relationship between Reactivity and Therapeutic Implications of These Therapeutic Modalities

Regardless of the types of cancer, NPs, and plasma devices, the combination of NPs and gas plasmas led to encouraging results. Regarding the mechanisms and effectiveness, current research is directed to the production of ROS/RNS and increase of NPs uptake. The currently available studies on combining NPs and gas plasma treatment in vitro and in vivo are summarized in Table 2.

Glioblastoma is the most studied tumor with a combination of gas plasmas and AuNPs, and numerous studies have emphasized the efficacy of concomitant treatments of these technologies compared to each of them. Increased cancer cell death, activation of tumor suppressors, inhibition of tumor growth, reduction of migration and invasion in cancer cells, increased induction of apoptosis, increased E-cadherin in treated tissues, and decreased tumor volume have been presented as a collection of the main findings. The action mechanisms were related to the production of ROS/RNS and enhancement of the uptake of NPs [154, 159–162].

Similar results for melanoma were obtained when gas plasmas and NPs were used together. The increase of ROS/RNS resulting from the combination of different configurations of gas plasma with FAK antibody conjugated-AuNPs, silica, silver, iron oxide, cerium oxide, titanium oxide, iron-doped titanium oxide NPs, and Anti-EGFR-AuNPs leads to a significant increase of early apoptosis and secondary necrosis, reduction in G2/M levels, increase in the sub-G1 fraction, FAS externalization, caspase-8 activation, increase of selective cancer cell death, inhibition the viability of cancer cells, and reduction of growth pattern [163–167].

In addition to the antiproliferative effects and induction of cytotoxic effects, decreased metastatic gene expression and increased cellular internalization of NPs have previously been revealed, where fluorouracil-loaded PLGA NPs and gas plasmas concomitantly are utilized as novel solutions for breast cancer oncotherapy [149]. Further, combined use of iron NPs and plasma jet exposure reduces cell proliferation and induces apoptosis and DNA fragmentation in breast cancer [168].

Iron oxide-based magnetic NPs and plasma jet treatment have previously been used to cause cell cycle arrest at the G0/G1 phase, apoptosis induction, condensation of nuclei, restraining tumor growth, intensive necrosis, and reduction of tumor size in lung cancer considering the high-level generation of ROS/RNS [169].

### 4.3. Challenges to Achieving Clinical Success and Future Needs

To avoid undesirable effects and target incurable tumors effectively, the mainstay strategy is combination therapies, which aims for cotreatments and integrating novel modalities with traditional methods. To this end, nanomedicine is combined with gas plasma as a multimodal and encouraging platform, as seen in the promising outcomes of preclinical studies. Based on these studies and the properties of gas plasma and NPs, the synergy of these two technologies can become an anticancer treatment strategy in the future. In particular, it is hoped that the synthesis of NPs with gas plasma or processing and subsequent plasma treatment will improve NPs in terms of preventing the degradation of conjugated drugs, delivery of optimum concentration and fluxes in desirable time, and improving the pharmacokinetics of the drug, which is consequently aimed to lead to enhanced cancer cell death and immunogenicity. Importantly, multifunctional and multimodal natures of NPs and gas plasma create a unique environment for cancer treatment.

Despite the increasing number of studies on cancer nanomedicine, there is a striking imbalance between preclinical and clinical applications, and the number of approved NPs, which are using for the clinical settings, is relatively limited [170]. Regarding plasma medicine, several devices have so far received accreditation as medical device class IIa in Europe [130]. However, plasma application in cancer patients has been mostly performed within exploratory studies [138, 171–175], and a guideline-based indication of plasma devices in cancer treatment is not given as of now. State-of-the-art NP synthesis methods, which can address some challenges of traditional bulk techniques, have not been considered in most clinical trials. Besides, a gap between preclinical and clinical studies indicates the need for additional clinical trials, especially given the acceptable safety profiles of some of the approaches (Table 1). As for the challenges facing plasma, since most research groups use self-made devices, the most critical issue is the lack of a specific framework for standardizing plasma devices. In addition, the multiplicity of factors involved in plasma processing and treatment causes obstacles to the definition of plasma dose. Therefore, more studies with the same treatment process and device are needed in addition to efforts to standardize and optimize these two technologies. An exciting option to combine both technologies would be to treat molecules or substances, including NPs, with gas plasma while the treated target has been modified to store the chemical energy of the short-lived plasma-derived ROS/RNS. At the delivery site in the TME, the highly reactive ROS/RNS modification can then perform the oxidative action to bring the gas plasma to the point of care via a detour.

Finally, there are future challenges for NP drug discovery and development in oncology. First, standardized procedures in production for research and commercial applications will help accelerate results translated from bench to bedside [176]. Second, organo-typic 3D cultures much more resemble the clinical pathology of cancer tissues than 2D cultures while being faster and more ethical animal models, potentially allowing for screening many different types of functionalized NPs, which will eventually allow propelling the field to clinically relevant approaches at higher speeds [177, 178]. Third, novel functionalization techniques such as genetically engineered cell membranes may aid the targeted delivery of NPs' cargo [179]. Last, preclinical research would vastly benefit from adhering to standardized protocols on studying NPs and assessing their drug delivery and distribution, as proposed by the National Cancer Institute (NCI) [179].

## 5. Conclusions

Nanomedicine and gas plasmas have been considered appropriate options for future oncotherapy due to the promising preliminary results. To improve nanomedicine's efficacy, combination with novel therapeutic modalities such as gas plasmas should be taken into account. Cocktail of ROS/RNS and electric fields of gas plasma and the NPs' ability to precisely targeting and penetrating tissues creates a putative oncotherapeutic platform for cancer treatment by enhancing selectivity and targeting chemotherapy resistance. Even though synergistic efficacy of NPs and gas plasmas are reported, more studies are essential to elucidate underlying mechanisms and impact on aggressive cancers.

## Figures and Tables

**Table 1:** Selected studies using nanoparticles in oncotherapy.

Tumor entity	Particle type	Main finding	Ref.
Preclinical studies			
Lung cancer	Polyurethane NPs, superparamagnetic iron oxide NPs coated with silica layers, mesoporous silica NPs, zinc oxide NPs, triphenylphosphonium-Pluronic F127 nanomicelles, cetuximab chitosan NPs, polymeric NPs, polyethyleneimine NPs coated with bovine serum albumin	Reduction in cancer cell survival, apoptosis induction (upregulating caspase-3, caspase-9, PARP, Bax), inhibition of lung tumor growth, pausing growth of cancerous cells, decrease in tumor size, induction of DNA leakage from nuclei by ROS/RNS, inhibition of metastasis, cell cycle arrest at G2/M phase, prevention of autophagy	[180–187]

Breast cancer	Porous silicon NPs, mesoporous maghemite NPs, PCE NPs, metal-organic frameworks, polymeric NPs (NVA-AA), porphyrin-based metal-organic framework carrier	Inhibition of metastasis; prevention of tumor growth; decrement of cell viability; suppression of cancer cell proliferation; reduction in tumor size; decrease in side effects; induction of apoptosis (downregulating Bcl-2 and upregulating caspase-3, UBA52, TIAL1, and PPP1C); suppression of cell motility and invasiveness; downregulating proteins involved in vesicular trafficking	[188–193]

Ovarian cancer	Selenium NPs, poly (lactic-co-glycolic) acid NPs with inorganic molybdenum octahedral cluster, Fe_2_O_3_ NPs, PEGL NPs, chitosan copolymer-magnetic NPs, poly-*ε*-caprolactone NPs	Inhibition of cancer cell growth, cytotoxic effect on cancer cells, reduction of metastasis, decrease of cancer cell viability and cytotoxicity, increased the intracellular ROS/RNS, diminution of tumor volume	[194–199]

Colon cancer	Albumin NPs, chitosan NPs, perfluorooctylbromide- porphyrin grafted lipid NPs, biosynthesized silver NPs, superparamagnetic iron oxide coated with mesenchymal stem cell, silver and gold NPs, mesoporous silica NPs coated with folic acid chitosan-glycine complex, hydroxyapatite NPs coated with gum Arabic, PLGA NPs co-loaded with 5-fluorouracil and perfluorocarbon	Enhancement of cancer cells killing; improved antitumor efficacy; prevention of tumor growth and metastasis; decrement of tumor volume; enhancement of photodynamic effects against cancer cells (by increasing oxidative stress); induction of apoptosis (overexpression of caspase-3, caspase-9, bid, and Bax); reduction of immune system response and systemic side effects; fragmentation of DNA in cancer cells; increase in antimitotic effects	[200–207]

Glioblastoma	Silver NPs, lanthanum oxide NPs, transferrin-conjugated porous silicon NPs, high-Z metal NPs, PEI surface-functionalized mesoporous silica NPs, PLGA NPs coated with polyvinyl alcohol and Poloxamer188, magnetic iron oxide NPs loaded trimethoxysilylpropyl-ethylenediamine triacetic acid, polymerized human serum albumin NPs, PEI-PEG-magnetic iron oxide NPs	Immense antitumor effect, increase in caspase activity, increase intrinsic and extrinsic apoptosis, diminution tumor cell viability, induce DNA damage and autophagic pathways, enhancing ROS/RNS, pausing cancer cell migration, causing a rupture of the lysosomal membranes, inhibition of cancer cell proliferation, downregulation of crucial enzymes for DNA repair and replication in cancer cells, upregulation of tumor suppressors	[208–214]

Pancreatic cancer	Magnetic NPs, nitric oxide donor S-nitroso-N-acetylpenicillamine loaded liposomes, PLGA NPs, polyanhydride NPs, solid lipid NPs, porous coordination network-Fe (III) NPs	Tumor growth inhibition, efficient tumor retention, enhancement of cytotoxicity; decrease of cell proliferation, reduction in cancer metastasis and progression, overexpression of proapoptotic genes, induction of ROS/RNS, improvement of anticancer treatment efficacy	[215–220]

Bone cancer	Superparamagnetic *γ*-Fe_2_O_3_ iron oxide with SiO2-CaO shell NPs, zinc oxide NPs; Fe ions-releasing mesoporous NPs, NPs with magnetic inner core and polymeric outer shell, alendronate-poly(amidoamine) NPs, metal-organic framework NPs	Increase of cytotoxicity in cancer cells, suppression of cancer cell growth, induction of apoptosis, exhibition of anticancer action, inhibition of the formation of osteoclasts, prevention of metastasis, induction of the polarization of tumor-resident macrophages to M1 phenotype	[221–226]

Prostate cancer	Selenium NPs, PLGA-PEG NPs, superparamagnetic iron oxide NPs, human serum albumin-coated NPs of (2) Ga, lipid-polymer hybrid NPs, manganese oxide–mesoporous silica, hexagonal boron nitride NPs	High anticancer activity, induction of tumor cell death via necrosis, increase of cytotoxicity, tumor regression, cell death induction, disruption of lysosomal structure in cancer cells, attenuation of lysosomal protease activity, modulator of autophagy	[227–232]
Liver cancer	Fe_3_O_4_-au nanoheterostructures, hydroxycamptothecin-based polyprodrug as the inner core, amphiphilic lipid-PEG as the outer shell NPs, exonanoRNA NPs, chondroitin-modified lipid NPs, glycogen NPs, rubber-like RNA NPs, CoFe_2_O_4_@MnFe_2_O_4_ magnetic NPs, mesoporous silica NPs	Significant cytotoxicity in cancerous cells, inhibition of tumor growth, induction of apoptosis, reduction in cell proliferation, increase of antitumor efficacy, inducing the enhanced permeability and retention effect, increment the release rate of the drug, reducing systemic side effects	[233–239]

Clinical trials			
Solid tumor in advanced stage	CYT-6091 (consist of AuNPs-PEG and tumor necrosis factor-*α*)	Treatment was well-tolerated, and one partial response was observed among 29 patients in this phase I study	[240, 241]

Colorectal cancer	CPX-1 (liposome-encapsulated formulation of irinotecan and floxuridine)	11 out of 13 patients showed disease control while 2 patients showed partial response	[242]

Breast cancer, lung cancer, colorectal cancer	FCE28068 (anthracycline doxorubicin linked to copolymers based on N-(2-hydroxypropyl) methacrylamide)	Response in breast and lung cancer patients, no response in colorectal cancer patients	[243]

Stomach cancer	MCC-465 (PEG immunoliposome-encapsulated doxorubicin)	Acute reactions related to infusion observed, no antitumor response observed, stable disease (SD) observed in 10 of 18 patients	[244]

Adenocarcinoma of the esophagus and gastroesophageal junction	SP1049C (doxorubicin in P-glycoprotein-targeting Pluronic)	9 out of 21 patients showed partial response, and 8 patients had either a minor response or stable disease	[245]

Advanced pancreatic cancer	Rexin-G (retroviral vector expressing a cytocidal cyclin G1 construct)	No antitumor activity observed	[246]

Pancreatic cancer	NK105 (a paclitaxel-incorporating micellar nanoparticle)	Partial response observed in 1 out of 11 patients, significant myelosuppression not observed up to 80 mg/m^−2^, pain or local toxicity in the area of the injection not observed in any patient, and 10 patients did not experience any hypersensitivity during the study	[247]

Pancreatic cancer	Lipoplatin (liposomal cisplatin) and gemcitabine	Partial response in 2/24 patients, disease stability in 14 patients, clinical benefit in 8 patients	[248]

**Table 2:** Studies on combining nanoparticle and gas plasma treatment in vitro and in vivo.

Particle and gas plasma type	Main finding	Tumor entity	Ref.
AuNPs and helium-based plasma jet	(i) Enhancement of the intracellular formation of superoxide andhydroxyl radical (ii) Decrease in intracellular glutathione (iii) Increase early apoptosis and secondary necrosis (iv) Caused a significant increase in the sub-G1 fraction (v) Reduction in G2/M levels (vi) FAS externalization and caspase-8 activation	Melanoma	[163]

AuNPs and plasma jet	(i) Increase of cell death (ii) Production of ROS/RNS (iii) decrease of cancer cells viability	Glioblastoma	[154]

Anti-NEU AuNPs and surface type air plasma	(i) Reduction of proliferation rate (ii) Increase of selective cancer cell death	Melanoma	[164]

PEG-coated AuNPs and surface DBD air plasma	(i) Decrease cancer cells viability (ii) Inhibiting tumor cell proliferation (iii) ROS/RNS-mediated apoptosis (iv) Activation of tumor suppressors (v) Inhibition of tumor growth (vi) Reduction of migration and invasion in cancer cells (vii) Decrease of tumor volume (viii) Increase of E-cadherin in treated tissues	Glioblastoma	[159]

AuNPs and DBD plasma	(i) Augmentation of anti-cancer cytotoxicity (ii) Increasing AuNP endocytosis and trafficking to lysosomes in cancer cells (iii) Enhancement of AuNP uptake	Glioblastoma	[160]

AuNPs and plasma jet	(i) Decrease of cell viability (ii) Improvement of NPs uptake rate into cells (iii) Increment of ROS/RNS intensity in the cancer cells	Glioblastoma	[161]

FAK antibody conjugated-AuNPs and DBD plasma	(i) Inhibition of the viability of cancer cells (ii) Induction of apoptosis (iii) Decrease of cell cycle phase in G1 (iv) Increase of the number of apoptotic cells	Melanoma	[165]

Fluorouracil loaded PLGA NPs and plasma jet	(i) Induction of cytotoxic effects (ii) Decrease of metastatic gene expression (iii) Enhancement of anti-cancer effects (iv) Exhibited anti-proliferative effects (v) Increase of cellular internalization of NPs	Breast cancer	[149]

Iron NPs and plasma jet	(i) Reduction in cell proliferation (ii) Induction apoptotic process (iii) Showed DNA fragmentation (iv) Increment of cancer cell death	Breast cancer	[168]

Epidermal growth factor conjugated AuNPs and DBD plasma	(i) Increase in cytotoxicity (ii) Enhancement of the apoptotic response	Lung cancer	[249]

Silica, silver, iron oxide, cerium oxide, titanium oxide, and iron-doped titanium oxide NPs, and plasma jet	(i) Reduction of growth pattern (ii) Increased cytotoxic effects (iii) ROS/RNS generation	Melanoma	[166]

AuNPs and plasma jet	(i) Increase of apoptotic cell death (ii) Induction of nuclear condensation and DNA fragmentation	Colorectal cancer	[250]
Iron oxide-based magnetic NPs and plasma jet	(i) Decrease of cell viability (ii) Indication of high levels of ROS/RNS (iii) G0/G1 Phase cell cycle arrest and condensation of nuclei (iv) Inhibitory effect on cell migration and invasion (v) Indicating intensive necrosis and apoptosis (vi) Inhibition of cancer cells proliferation (vii) Restraining tumor growth and reduction of tumor size	Lung cancer	[169]

PLGA-magnetic iron oxide NPs and plasma jet	(i) Inhibition of cancer cells proliferation (ii) Enhancement of cytotoxicity (iii) Induction of necrosis and apoptosis (iv) Increase of intracellular ROS/RNS levels	Lung cancer	[142]

Production of AuNPs by gas plasma	(i) Reduction of invasive cancer cell proliferation (ii) Induction of cancer cell apoptosis (iii) Impairment of cell migration	Breast cancer	[251]

Platinum NPs and plasma jet	(i) Decrease in the viability of cancer cells (ii) Enhancement the percentage of apoptosis cells (iii) Increment in the percentage of DNA fragmentation (iv) Decrease of cells in the G1 cell cycle phases (v) Induction of ROS/RNS production (vi) Augment of intracellular Ca^2+^ levels	Lymphoma	[252]

AuNPs and gas plasma	(i) Gas plasma-stimulated AuNP uptake (ii) Constant production of ROS/RNS (especially H_2_O_2_, NO_2_^−^, and NO_3_^−^) (iii) Gas plasma-induced lipid peroxidation (iv) Increase of AuNPs uptake through endocytosis	Glioblastoma	[162]

Anti-EGFR-AuNPs and air plasma	(i) Increment of death rate and proliferation (ii) Increase necrosis	Melanoma and oral cancer	[167]

PEG-AuNPs and plasma jet	(i) Production of singlet oxygen (ii) Hot electrons cause gold−PEG bond		[253]

Curcumin loaded on triphosphate chitosan NPs by plasma jet	(i) Decrease of cell viability (ii) Induction of sub-G1; arrest of G2/M (iii) Upregulation of TP53 mRNA expression as a tumor suppressor (iv) Increase in the percentage of apoptotic cells	Breast cancer	[254]

## Abbreviations

NPs: Nanoparticles
ROS: Reactive oxygen species
RNS: Reactive nitrogen species
ICD: Immunogenic cell death
TME: Tumor microenvironment
AuNPs: Gold nanoparticles
PTMs: Posttranslational modifications
PTS: Plasma-treated saline
TLR: Toll-like receptor
PEG: Polyethylenglycol
ASC: Adipose-derived stromal cells
PEI: Polyethyleneimine.
